# A phosphate and calcium-enriched diet promotes progression of 5/6-nephrectomy-induced chronic kidney disease in C57BL/6 mice

**DOI:** 10.1038/s41598-021-94264-8

**Published:** 2021-07-21

**Authors:** J. Radloff, N. Latic, U. Pfeiffenberger, C. Schüler, S. Tangermann, L. Kenner, R. G. Erben

**Affiliations:** 1 Department of Biomedical Sciences, University of Veterinary Medicine Vienna, Veterinaerplatz 1, 1210 Vienna, Austria; 2 Department of Pathobiology, University of Veterinary Medicine Vienna, Vienna, Austria

**Keywords:** Chronic kidney disease, Metabolic bone disease

## Abstract

C57BL/6 mice are known to be rather resistant to the induction of experimental chronic kidney disease (CKD) by 5/6-nephrectomy (5/6-Nx). Here, we sought to characterize the development of CKD and its cardiac and skeletal sequelae during the first three months after 5/6-Nx in C57BL/6 mice fed a calcium- and phosphate enriched diet (CPD) with a balanced calcium/phosphate ratio. 5/6-NX mice on CPD showed increased renal fibrosis and a more pronounced decrease in glomerular filtration rate when compared to 5/6-Nx mice on normal diet (ND). Interestingly, despite comparable levels of serum calcium, phosphate, and parathyroid hormone (PTH), circulating intact fibroblast growth factor-23 (FGF23) was 5 times higher in 5/6-Nx mice on CPD, relative to 5/6-Nx mice on ND. A time course experiment revealed that 5/6-Nx mice on CPD developed progressive renal functional decline, renal fibrosis, cortical bone loss, impaired bone mineralization as well as hypertension, but not left ventricular hypertrophy. Collectively, our data show that the resistance of C57BL/6 mice to 5/6-Nx can be partially overcome by feeding the CPD, and that the CPD induces a profound, PTH-independent increase in FGF23 in 5/6-Nx mice, making it an interesting tool to assess the pathophysiological significance of FGF23 in CKD.

## Introduction

Chronic kidney disease (CKD) is characterized by a decrease in glomerular filtration rate (GFR). The progressive loss of renal function is, over time, reflected in almost all major organ systems, as the kidney plays diverse roles throughout the body. CKD eventually leads to end-stage renal failure. However, a plethora of studies have shown that cardiovascular diseases associated with CKD are the primary cause of death in patients, even before they reach end-stage renal disease^1–3^.

CKD patients display chronically elevated levels of the phosphaturic hormone fibroblast growth factor-23 (FGF23). The rise in circulating intact FGF23 is tightly associated with declining kidney function and increased cardiovascular morbidity^4,5^. Until now, the exact cause of the increase in FGF23 serum levels after renal injury remains elusive, but accumulation due to declining GFR, reduced abundance of the co-receptor α-klotho (Klotho), anemia, release of specific metabolites from the injured kidney, and pro-inflammatory cytokines are being discussed as possible mechanisms^6–10^.

The so-called mineral and bone disorder (MBD) is common among patients with CKD. CKD-MBD results from disordered calcium and phosphorus regulation, in which the kidney plays a central role^11^. The loss of functional kidney mass together with the increase in circulating FGF23 suppress vitamin D activation in the kidney^12^. Low levels of the vitamin D hormone, as well as hyperphosphatemia induce parathyroid hormone (PTH) secretion. This form of secondary hyperparathyroidism is partially responsible for the development of CKD-MBD^11^.

CKD patients also often suffer from anemia, as erythropoietin levels drop and the accumulation of uremic substances leads to decreased clotting ability^13,14^. Recently, FGF23 signaling has also been directly linked to erythropoiesis and iron handling, further connecting CKD, MDB and cardiovascular disease^8^.

Multiple animal models are used to study and to understand the underlying interactions in the triangle of CKD, MBD and cardiovascular disease, but it has been difficult to find a model that reflects all three of these pathophysiological events. Due to their robustness and the availability of multiple genetically modified strains, C57BL/6 mice are one of the most used animal models in translational science. However, surgical induction of CKD via 5/6-nephrectomy (5/6-Nx) does not lead to the expected results in this mouse strain. The animals show only insignificant glomerulosclerosis, absent renal fibrosis, and normotension^15–17^. Furthermore, 5/6-Nx fails to induce an inflammatory response in C57BL/6 mice, which could be one of the driving factors behind the progression of CKD^18^. C57BL/6 mice also show increased resistance to CKD induction via albumin overload when compared to other strains^19^. The strain dependent resistance of C57BL/6 mice to CKD induction may involve low renin activity, since angiotensin II has been shown to overcome the resistance of C57BL/6 mice to 5/6-Nx^16,20^.

It is well known that increased phosphate intake facilitates the development and progression of renal injury in mice and men^3,21,22^. Moreover, hyperphosphatemia is also a major risk factor for cardiovascular disease, possibly bridging the gap between CKD and increased morbidity and mortality due to cardiovascular events^3^. Therefore, we hypothesized that increased phosphate intake would advance the progression of CKD in C57BL/6 mice. To increase dietary phosphate intake but to prevent the profound increase in PTH secretion associated with an isolated increase in dietary phosphate, we employed a calcium- and phosphate-rich diet (CPD) with a balanced calcium/phosphorus (Ca/P) ratio. After initially comparing the effects of the CPD and a normal mouse diet in 5/6-Nx C57BL/6 mice, we sequentially monitored renal, cardiovascular, and skeletal changes during the first three months after 5/6-Nx in C57BL/6 mice fed the CPD.

## Results

### Calcium and phosphate-enriched diet promotes 5/6 nephrectomy-induced renal functional impairment

**Figure 1:**
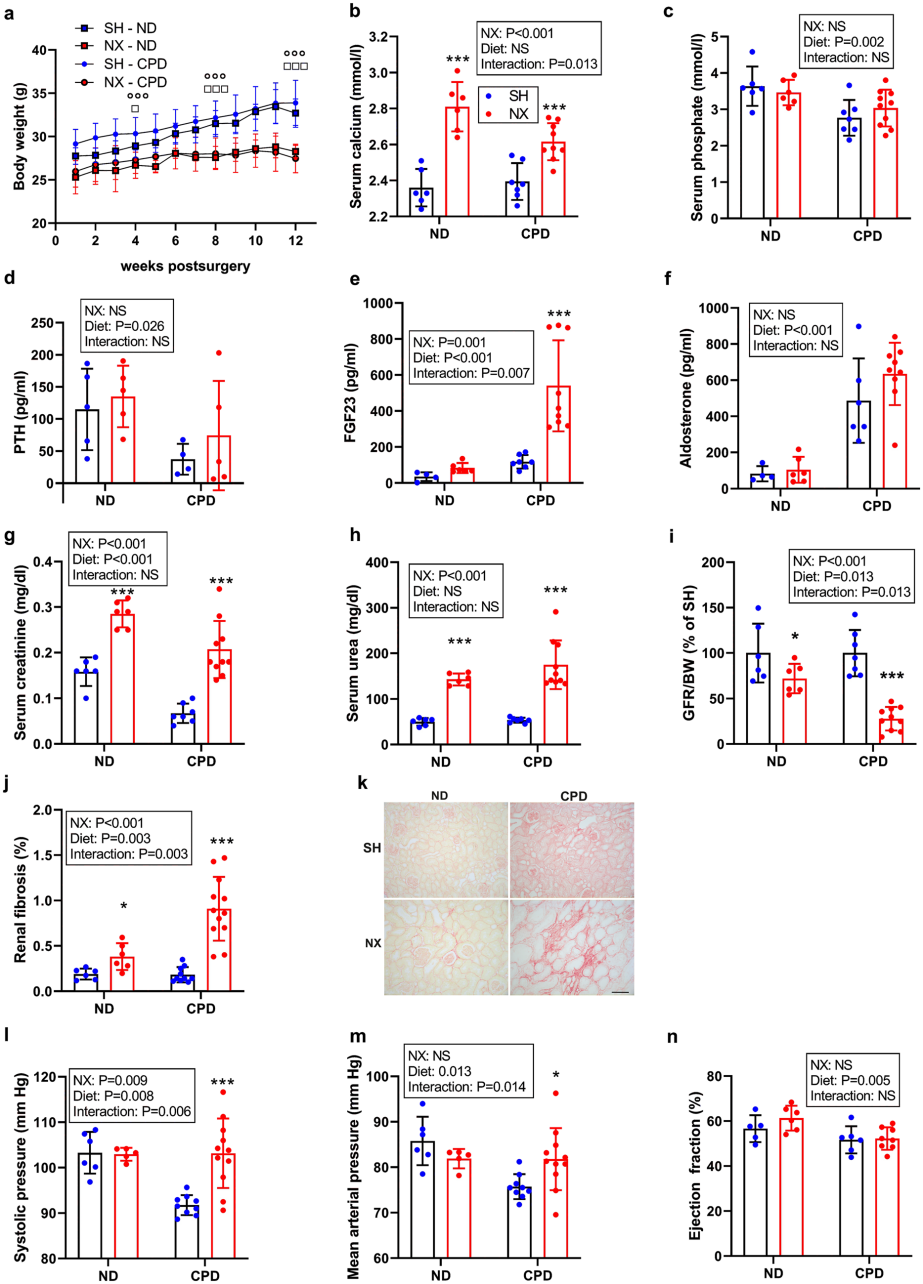
Calcium and phosphate-enriched diet promotes renal functional decline in 5/6-nephrectomized C57BL/6 mice. (**a**) Body weight, (**b**) serum calcium, (**c**) serum phosphate, (**d**) serum parathyroid hormone (PTH), (**e**) serum intact fibroblast growth factor-23 (FGF23), (**f**) serum creatinine, (**g**) serum urea, (**h**) glomerular filtration rate per g body weight (GFR/BW) expressed as percent of Sham control on same diet, (**i**) percent picrosirius red-stained area in kidney, (**j**) representative images of renal picrosirius red staining to assess renal fibrosis, (**k**) systolic and (**l**) mean arterial pressure measured by intraarterial catheterization, and (m) ejection fraction measured by echocardiography in sham-operated (Sham) and 5/6-Nx (NX) C57BL/6 mice on CPD and ND, 12 weeks postsurgery. N = 4–12 per group. Bars depict mean values ± SD. Data were analyzed using 2-way ANOVA, followed by Student’s *t* test. Insets show results of 2-way ANOVA. *P < 0.05; ***P < 0.001 versus Sham on same diet by Student’s *t* test. In (**a**) results of Student’s *t* test are shown only for the 4-, 8-, and 12-week time points (boxes, *P* < 0.05 vs. Sham on ND; circles, *P* < 0.001 vs. Sham on CPD). Bar = 100 µm in (**j**).

To examine the potential disease-promoting effects of a calcium and phosphate-enriched diet (CPD) on 5/6-Nx-induced CKD in C57BL/6 mice, we performed a pilot experiment in which we compared the effects of CPD with a normal mouse chow (normal diet, ND) on mineral homeostasis, kidney function, and cardiovascular function, 12 weeks postsurgery. The high lactose content of the CPD promotes paracellular uptake of minerals in the gut, resulting in enhanced vitamin D independent uptake of calcium and phosphate^23^. Both diets had the same energy content and nutrient distribution, and as a result body weight was influenced by surgery only. Sham mice were significantly heavier at the end of the 12-week period than 5/6-NX mice, independent of the diet they consumed (Fig. 1a). Surprisingly, both serum calcium and serum phosphate were not influenced by the diet (Fig. 1b,c). 5/6-NX mice were characterized by hypercalcemia, but did not show hyperphosphatemia, 12 weeks postsurgery (Fig. 1b,c). A key feature of the CPD is a balanced Ca/P ratio. Therefore, despite the high bioavailable phosphate content, the CPD did not cause an increase in serum intact PTH (Fig. 1d). Rather, serum PTH was suppressed by the CPD, likely due to the high calcium content. 5/6-Nx had only minor effects on PTH (Fig. 1d) but induced a distinct increase in circulating intact FGF23 that was profoundly modulated by the diet, i.e., 5/6-Nx mice showed a much more pronounced rise in intact FGF23 compared with Sham mice on the same diet (Fig. 1e). This is an interesting finding, because in contrast to an isolated increase in dietary phosphate, the CPD obviously dissociated between PTH and FGF23.

To assess renal function, we measured creatinine and urea levels in the serum, and kept the mice in metabolic cages overnight to assess GFR. Serum creatinine and urea levels were significantly increased in both 5/6-Nx groups (Fig. 1f,g). Although 2-way ANOVA did not show a significant interaction term, creatinine values were 1.8 times higher in 5/6-Nx mice on ND, and 3.1 times higher in 5/6-Nx mice on CPD when compared to the respective Sham groups (Fig. 1f). GFR as measured by creatinine clearance revealed a 40% decrease in 5/6-Nx mice on ND, but a 70% decrease in 5/6-Nx mice on CPD, 12 weeks postsurgery (Fig. 1h). In line with the more pronounced impairment in renal function in 5/6-Nx mice on CPD, CPD also significantly aggravated 5/6-Nx-induced renal fibrosis as measured by picrosirius red staining (Fig. 1i,j). To examine potential renal interstitial calcification we performed von Kossa staining of kidney sections. However, we found no evidence of calcium deposition in 5/6-Nx mice on both diets (Supplementary Figure S1). Cardiovascular monitoring by intra-arterial catheterization and echocardiography showed unchanged mean arterial pressure in 5/6-Nx mice on ND but increased systolic and mean arterial pressure in 5/6-Nx mice on CPD (Fig. 1k,l). However, cardiac function as assessed by echocardiographic measurement of ejection fraction remained unchanged in 5/6-Nx mice on both diets (Fig. 1m).

Based on the augmented renal functional impairment, increased circulating FGF23, increased renal fibrosis, and the presence of hypertension in 5/6-Nx mice on CPD compared with 5/6-Nx mice on ND, we decided to perform all subsequent experiments in mice maintained on CPD.

### 5/6 nephrectomy induced a progressive renal functional decline in C57Bl/6 mice on CPD

**Figure 2:**
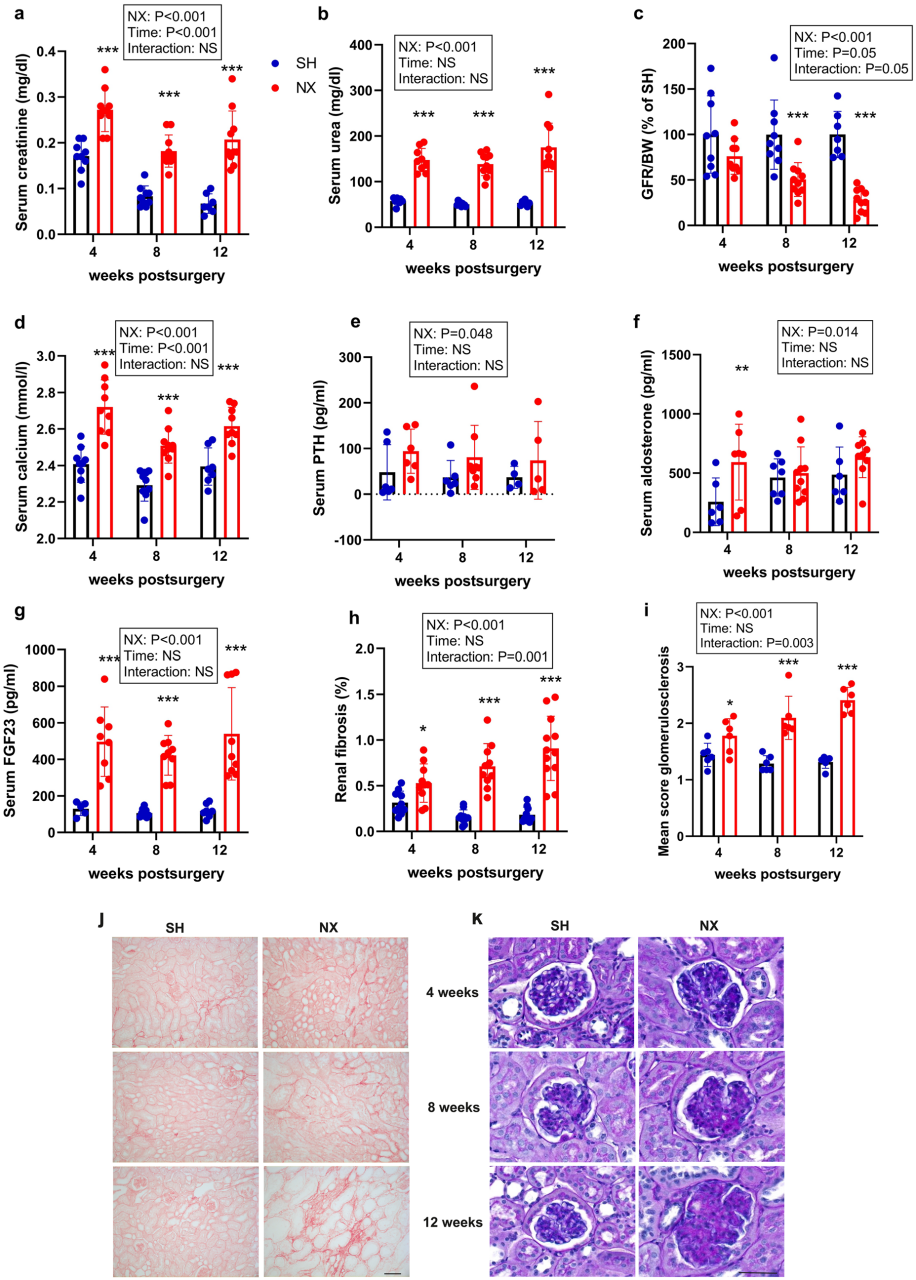
Progressive renal functional decline in 5/6-Nx mice on CPD. (**a**) Serum creatinine, (**b**) serum urea, (**c**) glomerular filtration rate per g body weight (GFR/BW) expressed as percent of Sham control at same time point, (**d**) serum calcium, (**e**) serum parathyroid hormone (PTH), (**f**) serum aldosterone, (**g**) serum intact fibroblast growth factor-23 (FGF23), (**h**) percent picrosirius red-stained area in kidney, (**i**) mean glomerulosclerosis score, (**j**) representative images of renal picrosirius red staining to assess renal fibrosis, and (**k**) representative images of renal PAS staining to assess glomerulosclerosis in Sham-operated (SH) and 5/6-Nx (NX) C57BL/6 mice on CPD, 4, 8 and 12 weeks postsurgery. N = 4–12 mice per group. Bars depict mean values ± SD. Data were analyzed using 2-way ANOVA, followed by Student’s t-test. Insets show results of 2-way ANOVA. *P < 0.05; **P < 0.01; ***P < 0.001 versus Sham at same time point by Student’s *t* test. Bar = 100 µm in (**j**) and 50 µm in (**k**), respectively.

To further establish this experimental CKD model, we performed a detailed time course experiment in mice fed the CPD. 5/6-Nx resulted in increased levels of creatinine and urea when compared to Sham animals throughout the experimental period (Fig. 2a,b). GFR as measured by creatinine clearance showed a time-dependent decline in 5/6-Nx mice, increasing from ~ 40% reduction at 4 weeks postsurgery to ~ 70% reduction at 12 weeks post-5/6-Nx (Fig. 2c). Taken together, these findings reflect progressive renal functional decline in 5/6-Nx animals on CPD.

Similar to our abovementioned pilot study, 5/6-Nx mice were characterized by hypercalcemia but did not show hyperphosphatemia, independent of the time postsurgery (Fig. 2d; Supplementary Fig. S2). Serum intact PTH and serum aldosterone showed moderate increases in 5/6-Nx mice at all time points (Fig. 2e,f). Serum potassium remained unchanged (Supplementary Fig. S2). In contrast, circulating intact FGF23 showed a distinct, about fivefold, increase vs. Sham mice, already at 4 weeks post-5/6-Nx, and remained at similar levels throughout the study period (Fig. 2g). Total serum iron levels were decreased in 5/6-Nx mice when compared to Sham at all time points, whereas serum cholesterol was increased (Supplementary Fig. S2).

In agreement with the well-known declining ability of the failing kidney to concentrate urine, 5/6-Nx animals produced significantly more urine than control animals, and urinary creatinine concentration was lower, compared with Sham controls (Supplementary Fig. S2). In addition, fractional urinary excretion of sodium, chloride, calcium, phosphate, and potassium were profoundly increased in 5/6-Nx mice throughout the study period (Supplementary Fig. S2).

To characterize morphological changes in the malfunctioning kidney, we assessed renal fibrosis via picrosirius red staining for collagen, and graded glomerulosclerosis in PAS-stained sections. The data revealed a time-dependent increase in interstitial fibrosis and glomerulosclerosis in 5/6-Nx mice (Fig. 2 h–k).

### Mild hypertension but absence of left ventricular hypertrophy and dysfunction in 5/6-Nx mice

**Figure 3:**
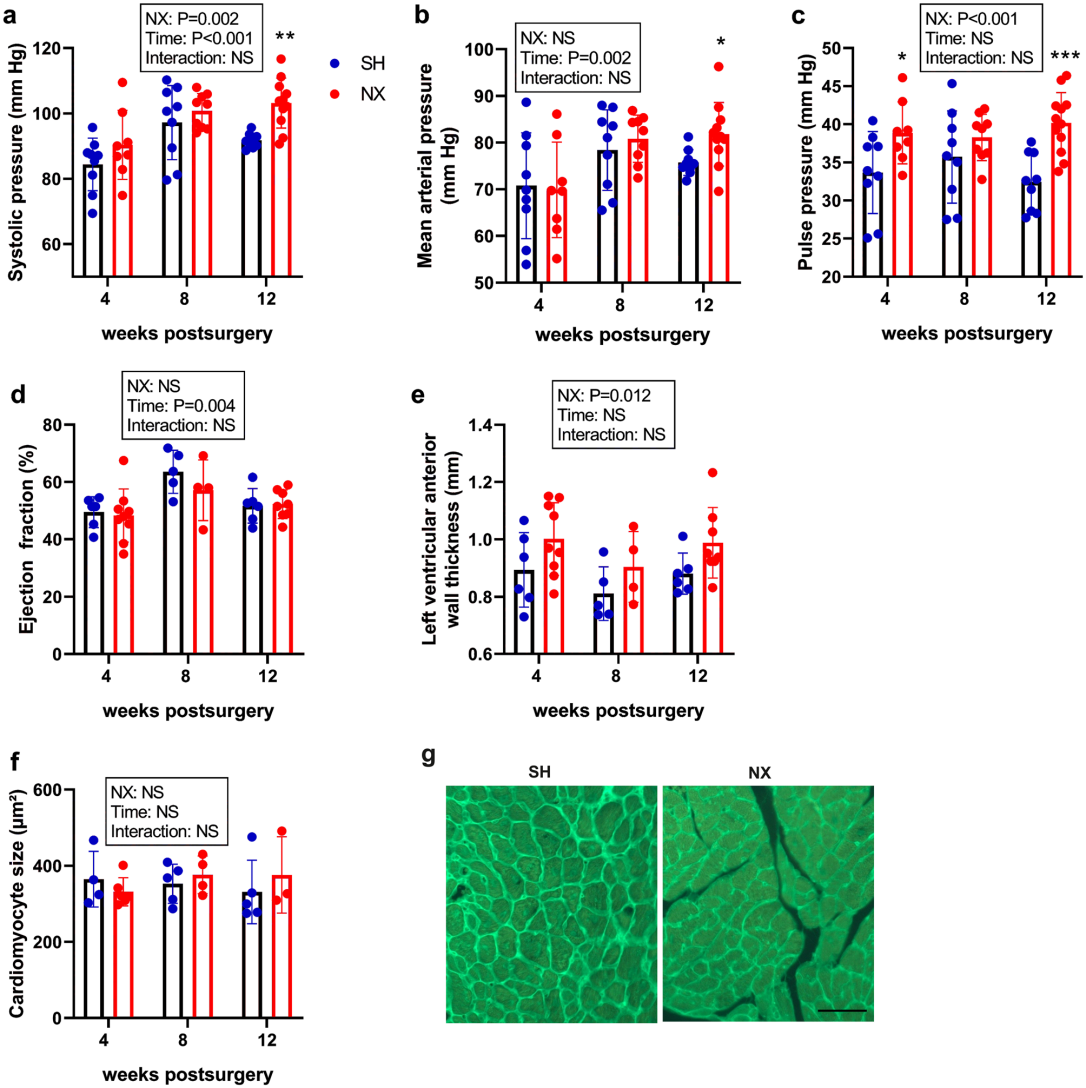
Mild hypertension but absence of left ventricular hypertrophy and dysfunction in 5/6-Nx mice on CPD. (**a**) Systolic, (**b**) mean arterial, and (**c**) pulse pressure measured by intraarterial catheterization, (**d**) ejection fraction and (**e**) left ventricular anterior wall thickness measured by echocardiography, and (**f**) cardiomyocyte size measured by (**g**) wheat germ agglutinin staining in Sham-operated (SH) and 5/6-Nx (NX) C57BL/6 mice on CPD, 4, 8 and 12 weeks postsurgery. N = 3–11 mice per group. Bars depict mean values ± SD. Data were analyzed using 2-way ANOVA, followed by Student’s *t* test. Insets show results of 2-way ANOVA. *P < 0.05; **P < 0.01; ***P < 0.001 versus Sham at same time point by Student’s *t* test. Bar = 50 µm in (**g**).

Multiple studies have shown that CKD is associated with higher risk for cardiovascular disease and overall mortality^1,24^. Therefore, we examined functional changes within the cardiovascular system in our murine CKD model by echocardiography, intraarterial and intracardial catheterization, and cardiac histological analysis. Intraarterial catheterization revealed unchanged blood pressure at 4 and 8 weeks post-5/6-Nx, but increased systolic and mean arterial pressure in 5/6-Nx at 12 weeks postsurgery, relative to Sham controls (Fig. 3a,b). The pulse pressure was increased in 5/6-Nx mice at 4 and 12 weeks (Fig. 3c), while heart rate, left ventricular contractility, and end-diastolic pressure were comparable between Sham and 5/6-Nx mice throughout the study (Supplementary Fig. S3). The lack of changes in end-diastolic pressure suggests that hypo- or hypervolemia were absent in 5/6-Nx mice. Echocardiography showed unchanged cardiac function as measured by ejection fraction and fractional shortening (Fig. 3d; Supplementary Fig. S3). Although echocardiography revealed slightly, but significantly increased diastolic left ventricular anterior wall thickness in 5/6-Nx mice, cardiomyocyte size as assessed by wheat germ agglutinin staining remained unchanged, and fibrosis as measured by picrosirius red staining was absent in histological sections of hearts from 5/6-Nx mice (Fig. 3e–g; Supplementary Fig. S3).

Collectively, these findings demonstrate mild hypertension at the later time points, but provide no evidence for left ventricular hypertrophy and/or dysfunction in 5/6-Nx mice on CPD.

### 5/6 nephrectomy induced reduced bone mass and impaired bone mineralization

**Figure 4:**
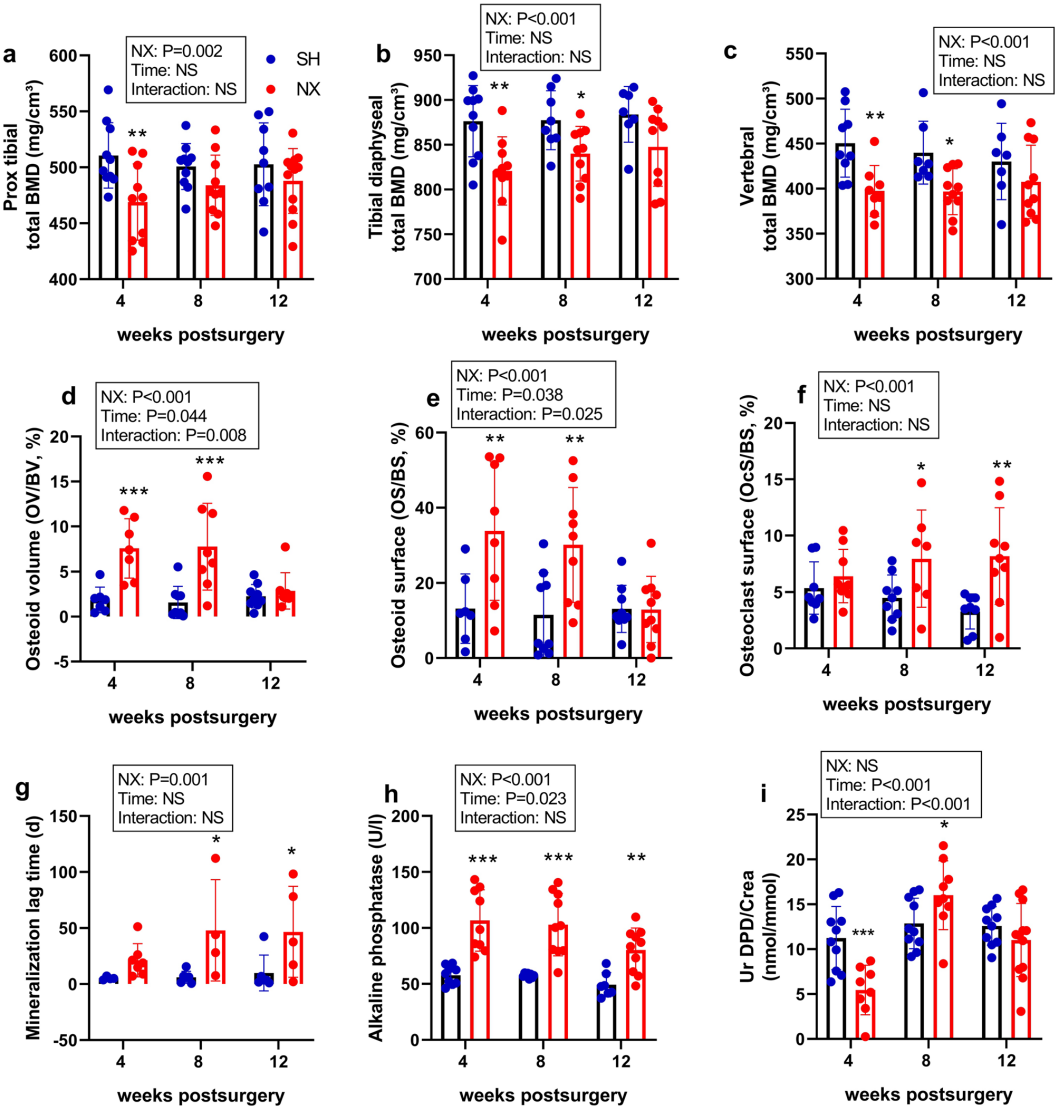
Reduced bone mass and impaired bone mineralization in 5/6-NX mice on CPD. (**a**) Proximal tibial total bone mineral density (BMD), (**b**) tibial diaphyseal total BMD, and (**c**) vertebral total BMD measured by pQCT; (**d**) osteoid volume, (**e**) osteoid surface, (**f**) osteoclast surface, and (**g**) mineralization lag time measured in tibial cancellous bone by histomorphometry; (**h**) serum alkaline phosphatase activity, and (**i**) urinary deoxypyridinoline/creatinine (UrDPD/Crea) excretion in Sham-operated (SH) and 5/6-Nx (NX) C57BL/6 mice on CPD, 4, 8 and 12 weeks postsurgery. N = 4–12 mice per group. Bars depict mean values ± SD. Data were analyzed using 2-way ANOVA, followed by Student’s *t* test. Insets show results of 2-way ANOVA. *P < 0.05; **P < 0.01; ***P < 0.001 versus Sham at same time point by Student’s *t* test.

To examine skeletal changes induced by 5/6-Nx in our model, we performed a diligent analysis of the bone phenotype, and assessed biochemical markers of bone turnover. pQCT analysis revealed a decrease in total volumetric BMD at the proximal tibia, the tibial shaft, and the lumbar spine in 5/6-Nx mice, relative to Sham controls (Fig. 4a–c). The 5/6-Nx-induced osteopenia was already evident at 4 weeks postsurgery and did not increase over time. At the tibial diaphysis, the 5/6-Nx-induced reduction in total BMD was associated with cortical thinning (Supplementary Fig. S4).

Histomorphometric analysis of tibial cancellous bone revealed an increase in osteoid volume in 5/6-Nx mice at 4 and 8 weeks, which returned to Sham levels at 12 weeks postsurgery (Fig. 4d). This was accompanied by an increase in osteoid surface at 4 and 8 weeks (Fig. 4e). Osteoclast surface was increased in 5/6-Nx mice at 8 and 12 weeks postsurgery (Fig. 4f). Although mineral apposition rate and bone formation rate remained unchanged in 5/6-Nx mice throughout the study (Supplementary Fig. S4), mineralization lag time was increased when compared to Sham controls at the later time points, demonstrating a subtle impairment in bone mineralization (Fig. 4g).

Furthermore, serum total alkaline phosphatase (AP) activity was significantly increased in 5/6-Nx mice at all time points when compared to Sham mice (Fig. 4h). In contrast to the elevated osteoclast numbers in 5/6-Nx mice at the later time points, whole body bone resorption as measured by urinary collagen crosslinks excretion was suppressed in 5/6-Nx mice at 4 weeks postsurgery and did not show consistent changes relative to Sham controls thereafter (Fig. 4i).

## Discussion

Although C57BL/6 mice are generally considered to be relatively resistant to the induction of CKD by 5/6-Nx^15–17^, we have shown here that 5/6-Nx C57BL/6 mice on a calcium and phosphate-enriched diet develop a ~ 70% reduction in creatinine clearance associated with mild hypertension and impaired bone mineralization, 12 weeks postsurgery.

CKD globally affects 11 to 13% of the population^25^. Diabetes is the main cause of CKD, while other diseases such as glomerulonephritis, genetic disorders such as polycystic kidney disease, drug-induced renal injury and ascending urinary tract infections can ultimately also lead to CKD^26^. Since the causes are so diverse it has been difficult to create a translational model representing all clinically relevant properties of CKD. 5/6-Nx is often considered the gold standard of experimental CKD models because of the slowly progressing disease process and the absence of any toxic substances inducing renal damage. Toxic substances that have frequently been used in experimental murine CKD models include adenine, folic acid, or aristolochic acid^27–29^. However, a general drawback of the latter methods is that they are driven by the specific pathologies induced by the respective substance used.

C57BL/6 mice have been shown to be rather resistant to 5/6-induced CKD when compared to other strains^16,17,19,30^. To increase renal injury, 5/6-Nx has been combined with other methods, such as unilateral clamping or implantation of angiotensin II-releasing minipumps. Unilateral clamping resulted in progressive loss of renal function, but is not suitable for longer studies, as the overall survival rate dropped to 80% after 12 weeks^31^. In addition, the ischemic injury model is rather delicate as it requires exact timing of clamping duration and tight body temperature monitoring. Constant application of angiotensin II via subcutaneous minipumps in combination with 5/6-Nx is another established method to induce renal functional decline and glomerulosclerosis in C57BL/6 mice^16^.

In this study we showed that 5/6-Nx C57BL/6 mice on a calcium and phosphate-enriched diet developed significant azotemia, progressive decline in GFR, glomerulosclerosis, and interstitial renal fibrosis. Important features of the CPD used in this study is a balanced Ca/P ratio and the high lactose content, facilitating paracellular intestinal uptake of minerals. This resulted in a suppression of PTH secretion, but an upregulation of circulating intact FGF23. In addition, 5/6-Nx induced an over-additive increase in intact FGF23 in mice on CPD. This may be an interesting finding, because circulating intact FGF23 is currently being discussed as a disease-promoting factor in CKD patients^32,33^. Moreover, the rise in serum FGF23 occurred in the complete absence of hyperphosphatemia in 5/6-Nx mice on either ND or CPD, further corroborating the notion that the early increase in FGF23 in CKD patients occurs independent of hyperphosphatemia and elevated PTH^6^. The mechanism underlying the CKD-induced rise in circulating FGF23 is currently unknown. However, because serum levels of calcium and phosphate were similar in 5/6-Nx mice on ND and CPD, our data suggest that these factors are likely not the driving factors behind augmented FGF23 secretion in CKD.

Interestingly, renal fibrosis in 5/6-Nx mice on ND was not significantly increased when compared to Sham controls, whereas the increase in renal fibrosis was distinct in 5/6-Nx mice on CPD. Although the molecular mechanism is unknown, this finding may point into the direction that increased dietary phosphate accelerates renal fibrosis in a pre-injured kidney. A recent study has shown that 2% of dietary phosphate leads to significant nephron loop disorganization after 8 weeks and calcium depositions after 12 weeks in C57BL/6 wild-type mice^34^. Furthermore, mice that were kept on a high phosphate diet for 12 weeks also showed significant fibrosis even without preexisting kidney injury^34^. Conversely, application of the phosphate binder ferric citrate reduced collagen expression levels and renal fibrosis in a genetic model of CKD (*Col4a3* knockout) on a C57BL/6 background^35^. Although further work needs to be done to substantiate this hypothesis, it is tempting to speculate that excessive FGF23 may be the missing link between dietary phosphate and renal fibrosis in our model.

A limitation of our model is that we did not observe major cardiovascular effects apart from increased pulse pressure, a slight increase in diastolic left ventricular anterior wall thickness, and mild hypertension in 5/6-Nx mice on CPD at the end of the study. Histological assessment did not provide any evidence for left ventricular hypertrophy or cardiac fibrosis in 5/6-Nx mice. It is likely that the increase in blood pressure can be explained, at least partially, by an activated renin–angiotensin–aldosterone system, as aldosterone levels were significantly higher in 5/6-Nx mice when compared to Sham controls. We did not observe structural cardiovascular adaptions to chronically increased blood pressure in our model up to 12 weeks postsurgery. In agreement with our findings, cardiovascular effects could only be seen after 20 weeks in an adenine-induced murine CKD model^36^. Therefore, it may be necessary to examine later time points in our model to see structural cardiovascular changes. The increased pulse pressure in 5/6-Nx mice might be explained by anemia. We did not assess anemia in our study. However, previous studies have shown that 5/6-Nx leads to anemia in C57BL/6 mice^37^. In this context it has been reported that anemia can cause increased pulse pressure due to a decrease in blood viscosity^38^.

Circulating FGF23 is associated with left ventricular hypertrophy and overall higher cardiovascular morbidity and mortality in CKD patients^4,5,39^. A still unresolved question in this respect is whether FGF23 is just a good biomarker or whether there is a causal link between FGF23 and cardiovascular disease. Substantial evidence has been provided in favor of a direct pro-hypertrophic effect of FGF23 on the heart. Faul et al. showed that treatment of cultured rat cardiomyocytes with FGF23 leads to hypertrophy, and that FGF23 injection induces left ventricular hypertrophy in rodents^40^. In addition, FGF23 suppression by the calcimimetic etelcalcetide inhibited progression of left ventricular hypertrophy in patients on hemodialysis, also supporting a direct pro-hypertrophic action of FGF23^41^. On the other hand, chronically elevated circulating intact FGF23 does not lead to left ventricular hypertrophy in *Hyp* mice, a murine model of X-linked hypophosphatemia^42^. Moreover, in the current study we found no evidence for left ventricular hypertrophy or cardiac fibrosis in 5/6-Nx mice despite high levels of circulating FGF23. Hence, it is possible that the direct pro-hypertrophic effect of FGF23 in the heart is context-dependent.

CKD patients often suffer from MBD. Therefore, the characterization of skeletal effects in 5/6-Nx mice on CPD was one of the endpoints of this study. The major symptoms of MBD are bone pain, increased fracture risk, disordered calcium and phosphate metabolism, and peripheral calcification^11^. 5/6-Nx mice showed hypercalcemia and increased levels of serum alkaline phosphatase throughout the study. Serum alkaline phosphatase can be a biomarker for increased bone metabolism, and is associated with mortality in dialysis patients^43^. pQCT analysis showed 5/6-Nx-induced loss of volumetric BMD in long bones and the axial skeleton. Low BMD is a risk factor for bone fractures in patients with CKD, and therefore clinically highly relevant^44^. Histomorphometric analysis revealed a transient increase in osteoid volume and osteoid surface at 4 and 8 weeks post-5/6-Nx. These changes were accompanied by an increase in osteoclast numbers and in mineralization lag time in 5/6-Nx mice at 8 and 12 weeks postsurgery. We reported earlier that the increase in osteocytic FGF23 secretion locally contributes to the impairment in bone mineralization in 5/6-Nx mice^45^. In addition, secondary hyperparathyroidism is likely to be a major factor causing increased bone turnover in CKD. The mechanisms leading to secondary hyperparathyroidism in CKD include not only the reduction in functional kidney mass and FGF23-induced suppression of vitamin D hormone synthesis, but also skeletal resistance to PTH. In this context, it has been shown that accumulation of the uremic toxin indoxyl sulfate decreases PTH receptor expression in osteocytes^46^.

In conclusion, here we present a 5/6-Nx-induced model of CKD in C57BL/6 mice on a calcium and phosphate-enriched diet that leads to progressive renal functional decline together with progressive renal fibrosis and glomerulosclerosis, and that recapitulates some typical features of MBD such as hypercalcemia, secondary hyperparathyroidism, and low BMD in the axial and appendicular skeleton.

## Materials and methods

### Animals and diets

The study was undertaken in accordance with prevailing EU and national guidelines for animal care and welfare, and in compliance with ARRIVE guidelines. All animal procedures were approved by the Ethical Committees of the University of Veterinary Medicine Vienna and of the Austrian Federal Ministry of Education, Science and Research.

Male C57BL/6N mice were raised on a standard mouse chow (normal diet, ND) (Sniff™), containing 1% calcium and 0.7% phosphorus, and were switched to a calcium and phosphate-rich diet (CPD) containing 2.0% calcium, 1.25% phosphorus, and 20% lactose at least two weeks before surgery. The high lactose concentration of the diet facilitates paracellular intestinal calcium and phosphate uptake in a vitamin D independent fashion^23^. The Ca/P ratio of diets was similar, 1.43 for ND and 1.60 for CPD. Both the CPD and the ND had the same energy content (14 MJ/kg) and the same nutrient distribution (carbohydrates, protein, fat). For the duration of the experiments all animals were kept in pairs of 2 at 22–24 °C and a 12 h light/12 h dark cycle with ad libitum access to water and food. Before necropsy, urine was collected overnight in metabolic cages. To assess the progression of CKD, 10 to 12 mice per group were killed at 4, 8 and 12 weeks postsurgery after in vivo double calcein labeling (20 mg/kg) on days 9 and 6 before necropsy.

### 5/6-nephrectomy model

CKD was induced via 5/6-Nx in a two-step procedure. Anesthesia was initiated by intraperitoneal injection of ketamine and medetomidine (17/0.08 mg/kg) and maintained by isoflurane (2%). Pain management before surgery included the subcutaneous application of buprenorphine and metamizole. In addition, the animals were given metamizole for 3 days postsurgery and buprenorphine only after careful consideration of each animal’s status. Post-surgical assessment was carried out once a day, and mice were kept on heating mats to aid recovery. For initial 2/3 nephrectomy, the left kidney was exposed via flank incision. Employing a surgical microscope, the kidney was decapsulated to prevent ureter damage. Upper and lower kidney poles were cut, while the renal arteria and vein were clamped for a maximum of 90 s to prevent blood loss on one hand, and acute kidney injury on the other. Electrocautery was performed on both sides of the remnant kidney and a hemostatic collagen membrane was applied (Gelaspon, Chauvin Ankerpharm, Berlin, Germany). Right kidney nephrectomy was carried out after one week of recovery. Removed kidney tissue was weighed to ensure proper 5/6-Nx. Sham surgery was also performed as a two-step procedure in which the respective kidney was exposed and then repositioned. Skin incisions were closed with single sutures.

### Biochemical analyses

For biochemical analysis of serum and urine samples we employed a Cobas c111 autoanalyzer (Roche, Basel, Switzerland). Serum intact PTH (Immutopics), serum intact FGF23 (Kainos) and serum aldosterone (NovaTec) were determined by ELISA.

### Transthoracic Doppler echocardiography (ECG)

Echocardiography was performed two to four days before necropsy, using a 14 MHz linear transducer (Siemens Accuson s2000) under 1% isoflurane anesthesia. Anatomic M-mode recorded in the short axis view at the papillary muscles level was used to assess left ventricular (LV) wall thickness, internal dimensions, and fractional shortening. LV diastolic function was evaluated using diastolic flow through the mitral valve, employing pulsed-wave Doppler in the apical 4-chamber view. To ensure proper analysis, a minimum of 5 cardiac cycles were averaged for each parameter.

### Central arterial pressure measurements and cardiac catheterization

Aortic and cardiac pressures were measured to assess cardiac function. A SPR-671NR pressure catheter (1.4 F, Millar Instruments, Houston, TX, USA) was inserted into the carotid artery under general anesthesia (2% isoflurane). Measurements were carried out at 1.5%. Later, the catheter was pushed forward into the left ventricle for the measurement of cardiac parameters. To ensure proper data analysis, pressure traces were recorded over 5 min and analyzed using LabChart7 software.

### Histological evaluation of renal fibrosis, calcification, and glomerulosclerosis

Renal tissue samples were fixed in 4% paraformaldehyde, and after routine processing embedded in paraffin. Sections of 5 µm were cut and stained according to standard protocols for Picrosirius Red (PSR), Periodic Acid–Schiff (PAS), and von Kossa. Fibrosis was assessed using ImageJ on PSR-stained sections, whereas glomerulosclerosis was quantified in PAS-stained sections. Mesangial expansion was graded in 20 randomly chosen glomeruli per animal, ranging from 1 (< 25%) to 4 (> 75%). Based on these data, a mean glomerulosclerosis score was calculated for each animal.

### Histological evaluation of cardiac fibrosis and cardiomyocyte hypertrophy

Hearts were processed as described for the kidney and stained with picrosirius red. Fibrotic tissue from the whole circumference of the left ventricle and septum at the level of the papillary muscles was quantified using ImageJ and expressed as % stained area. For the assessment of cardiomyocyte size, sections were stained with FITC-labeled wheat germ agglutinin. Cardiomyocyte size was analyzed using a semi-automated procedure from various regions of the left ventricle and septum, employing ImageJ.

### Bone mineral density (BMD) measurements

Volumetric BMD was measured by peripheral quantitative computed tomography (pQCT) using an XCT Research M + pQCT machine (Stratec Medizintechnik, Pforzheim, Germany) and a voxel size of 70 µm. Measurements for the tibia were carried out in the shaft (2 mm proximal to the tibiofibular junction) and the proximal metaphysis (1.5, 2.0 and 2.5 mm distal to the proximal tibial growth plate). For the L3 vertebra, three slices were measured and averaged, one in a mid-transversal plane, and two located 0.5 mm rostral and caudal of the mid-transversal plane. To discriminate between trabecular and cortical bone in both tibia and L3, a threshold of 450 mg/cm^3^ was used. A threshold of 600 mg/cm^3^ was used for calculation of cortical BMD at the tibial shaft.

### Bone histology and histomorphometry

Femurs were fixed in 4% paraformaldehyde at 4 °C overnight and were processed and embedded in methylmethacrylate as described previously^47^. Midsagittal sections of the distal femurs were prepared using a HM 355S microtome (Microm, Walldorf, Germany) and were stained with von Kossa/McNeal^47^. Histomorphometric measurements were made on sections stained with von Kossa/McNeal using a semiautomatic system (Osteomeasure, Osteometrics, Decatur, GA, USA) and a Zeiss Axioskop microscope (Carl Zeiss Microscopy, Jena, Germany) with a drawing attachment. The area within 0.25 mm from the growth plate was excluded from the measurements.

### Statistical analysis

Statistical analysis was carried out with GraphPad Prism version 9. Two-way ANOVA was calculated using the fixed factors of diet, time, and surgery, followed by Student’s t-test for individual comparisons between Sham and 5/6-Nx groups at specific time points or on specific diets. Data are presented as mean ± standard deviation. P values ≤ 0.05 were considered significant.

## Supplementary Information

Supplementary Information.

## Data Availability

All data generated or analyzed in this study are included in this article (and its supplementary information files).

## References

[1] Go AS, Chertow GM, Fan D, McCulloch CE, Hsu C (2004). Chronic kidney disease and the risks of death, cardiovascular events, and hospitalization. N. Engl. J. Med..

[2] Ronco C, Bellasi A, Di Lullo L (2018). Cardiorenal syndrome: An overview. Adv. Chronic Kidney Dis..

[3] Kendrick J, Kestenbaum B, Chonchol M (2011). Phosphate and cardiovascular disease. Adv. Chronic Kidney Dis..

[4] Kendrick J (2011). FGF-23 associates with death, cardiovascular events, and initiation of chronic dialysis. JASN.

[5] Seiler S (2014). Associations of FGF-23 and sKlotho with cardiovascular outcomes among patients with CKD stages 2–4. Clin. J. Am. Soc. Nephrol..

[6] Isakova T (2011). Fibroblast growth factor 23 is elevated before parathyroid hormone and phosphate in chronic kidney disease. Kidney Int..

[7] Olauson H, Larsson TE (2013). FGF23 and Klotho in chronic kidney disease. Curr. Opin. Nephrol. Hypertens..

[8] Noonan ML (2020). Erythropoietin and a hypoxia-inducible factor prolyl hydroxylase inhibitor (HIF-PHDi) lowers FGF23 in a model of chronic kidney disease (CKD). Physiol. Rep..

[9] Egli-Spichtig D (2019). Tumor necrosis factor stimulates fibroblast growth factor 23 levels in chronic kidney disease and non-renal inflammation. Kidney Int..

[10] Simic P (2020). Glycerol-3-phosphate is an FGF23 regulator derived from the injured kidney. J. Clin. Investig..

[11] Waziri B, Duarte R, Naicker S (2019). Chronic kidney disease—Mineral and bone disorder (CKD-MBD): Current perspectives. Int. J. Nephrol. Renovasc. Dis..

[12] Shimada T (2004). FGF-23 is a potent regulator of vitamin D metabolism and phosphate homeostasis. J. Bone Miner. Res..

[13] Boccardo P, Remuzzi G, Galbusera M (2004). Platelet dysfunction in renal failure. Semin. Thromb. Hemost..

[14] Babitt JL, Lin HY (2012). Mechanisms of anemia in CKD. JASN.

[15] Kren S, Hostetter TH (1999). The course of the remnant kidney model in mice. Kidney Int..

[16] Leelahavanichkul A (2010). Angiotensin II overcomes strain-dependent resistance of rapid CKD progression in a new remnant kidney mouse model. Kidney Int..

[17] Ma L-J, Fogo AB (2003). Model of robust induction of glomerulosclerosis in mice: importance of genetic background. Kidney Int..

[18] Furusho T (2020). Renal TNFα activates the WNK phosphorylation cascade and contributes to salt-sensitive hypertension in chronic kidney disease. Kidney Int..

[19] Ishola DA (2006). In mice, proteinuria and renal inflammatory responses to albumin overload are strain-dependent. Nephrol. Dial. Transplant..

[20] Nogueira A, Pires MJ, Oliveira PA (2017). Pathophysiological mechanisms of renal fibrosis: A review of animal models and therapeutic strategies. Vivo.

[21] Lau WL (2013). High phosphate feeding promotes mineral and bone abnormalities in mice with chronic kidney disease. Nephrol. Dial. Transplant..

[22] Ritter CS, Slatopolsky E (2016). Phosphate toxicity in CKD: The killer among us. Clin. J. Am. Soc. Nephrol..

[23] Kollenkirchen U, Fox J, Walters MR (1991). Normocalcemia without hyperparathyroidism in vitamin D-deficient rats. J. Bone Miner. Res..

[24] Ku E, Lee BJ, Wei J, Weir MR (2019). Hypertension in CKD: Core curriculum 2019. Am. J. Kidney Dis..

[25] Hill NR (2016). Global prevalence of chronic kidney disease—A systematic review and meta-analysis. PLoS ONE.

[26] Evans PD, Taal MW (2011). Epidemiology and causes of chronic kidney disease. Medicine.

[27] Jia T (2013). A novel model of adenine-induced tubulointerstitial nephropathy in mice. BMC Nephrol..

[28] Long DA, Woolf AS, Suda T, Yuan HT (2001). Increased renal angiopoietin-1 expression in folic acid-induced nephrotoxicity in mice. J. Am. Soc. Nephrol..

[29] Yang, L., Besschetnova, T. Y., Brooks, C. R., Shah, J. V. & Bonventre, J. V. Epithelial cell cycle arrest in G2/M mediates kidney fibrosis after injury. *Nat Med* **16**, 535–543 (2010). 10.1038/nm.2144 PMC3928013 20436483

[30] Hamzaoui M (2020). 5/6 nephrectomy induces different renal, cardiac and vascular consequences in 129/Sv and C57BL/6JRj mice. Sci. Rep..

[31] Wei J (2019). New mouse model of chronic kidney disease transitioned from ischemic acute kidney injury. Am. J. Physiol. Renal Physiol..

[32] Singh S (2016). Fibroblast growth factor 23 directly targets hepatocytes to promote inflammation in chronic kidney disease. Kidney Int..

[33] Komaba H, Fukagawa M (2012). The role of FGF23 in CKD–with or without Klotho. Nat. Rev. Nephrol..

[34] Shen Z-J, Hu J, Shiizaki K, Kuro-o M, Malter JS (2016). Phosphate-induced renal fibrosis requires the prolyl isomerase pin1. PLoS ONE.

[35] Francis C (2019). Ferric citrate reduces fibroblast growth factor 23 levels and improves renal and cardiac function in a mouse model of chronic kidney disease. Kidney Int..

[36] Kieswich JE (2018). A novel model of reno-cardiac syndrome in the C57BL/ 6 mouse strain. BMC Nephrol..

[37] Yokoro M (2017). Asymmetric dimethylarginine contributes to the impaired response to erythropoietin in CKD-anemia. JASN.

[38] Tanimura, M. et al. Effect of anemia on cardiovascular hemodynamics, therapeutic strategy and clinical outcomes in patients with heart failure and hemodynamic congestion. *Circ. J.* **81**, 1670–1677 (2017). 10.1253/circj.CJ-17-0171 28626160

[39] Foley RN (1995). Clinical and echocardiographic disease in patients starting end-stage renal disease therapy. Kidney Int..

[40] Faul C (2011). FGF23 induces left ventricular hypertrophy. J. Clin. Investig..

[41] Dörr K (2021). Randomized trial of etelcalcetide for cardiac hypertrophy in hemodialysis. Circ. Res..

[42] Liu ES (2018). Increased circulating FGF23 does not lead to cardiac hypertrophy in the male hyp mouse model of XLH. Endocrinology.

[43] Regidor DL (2008). Serum alkaline phosphatase predicts mortality among maintenance hemodialysis patients. JASN.

[44] West SL (2015). Bone mineral density predicts fractures in chronic kidney disease. J. Bone Miner. Res..

[45] Andrukhova O, Schüler C, Bergow C, Petric A, Erben RG (2018). Augmented fibroblast growth factor-23 secretion in bone locally contributes to impaired bone mineralization in chronic kidney disease in mice. Front. Endocrinol. (Lausanne).

[46] Nii-Kono T (2007). Indoxyl sulfate induces skeletal resistance to parathyroid hormone in cultured osteoblastic cells. Kidney Int..

[47] Erben RG, Glösmann M (2012). Histomorphometry in rodents. Methods Mol. Biol..

