# Supplementary material for: A phosphate and calcium-enriched diet promotes progression of 5/6-nephrectomy-induced chronic kidney disease in C57BL/6 mice
Source: Sci Rep. 2021 Jul 21;11:14868. doi: 10.1038/s41598-021-94264-8 (PMC8295299; doi:10.1038/s41598-021-94264-8)
Supplement: Supplementary file 1 — Supplementary Information. [file 41598_2021_94264_MOESM1_ESM.pdf]

Supplementary material

A phosphate and calcium-enriched diet promotes progression of 5/6-nephrectomy-induced chronic kidney disease in C57BL/6 mice

J Radloff[1], N Latic[1], U Pfeiffenberger[1], C Schüler[1], S Tangermann[2], L Kenner[2], RG Erben[1*]

***Corresponding author:**

Reinhold G. Erben, M.D., D.V.M.

Institute of Physiology, Pathophysiology and Biophysics

Dept. of Biomedical Sciences

University of Veterinary Medicine Vienna

Veterinaerplatz 1, 1210 Vienna, Austria

Phone +43-1-250 77 4550, Fax +43-1-250 77 4599

E-mail Reinhold.Erben@vetmeduni.ac.at

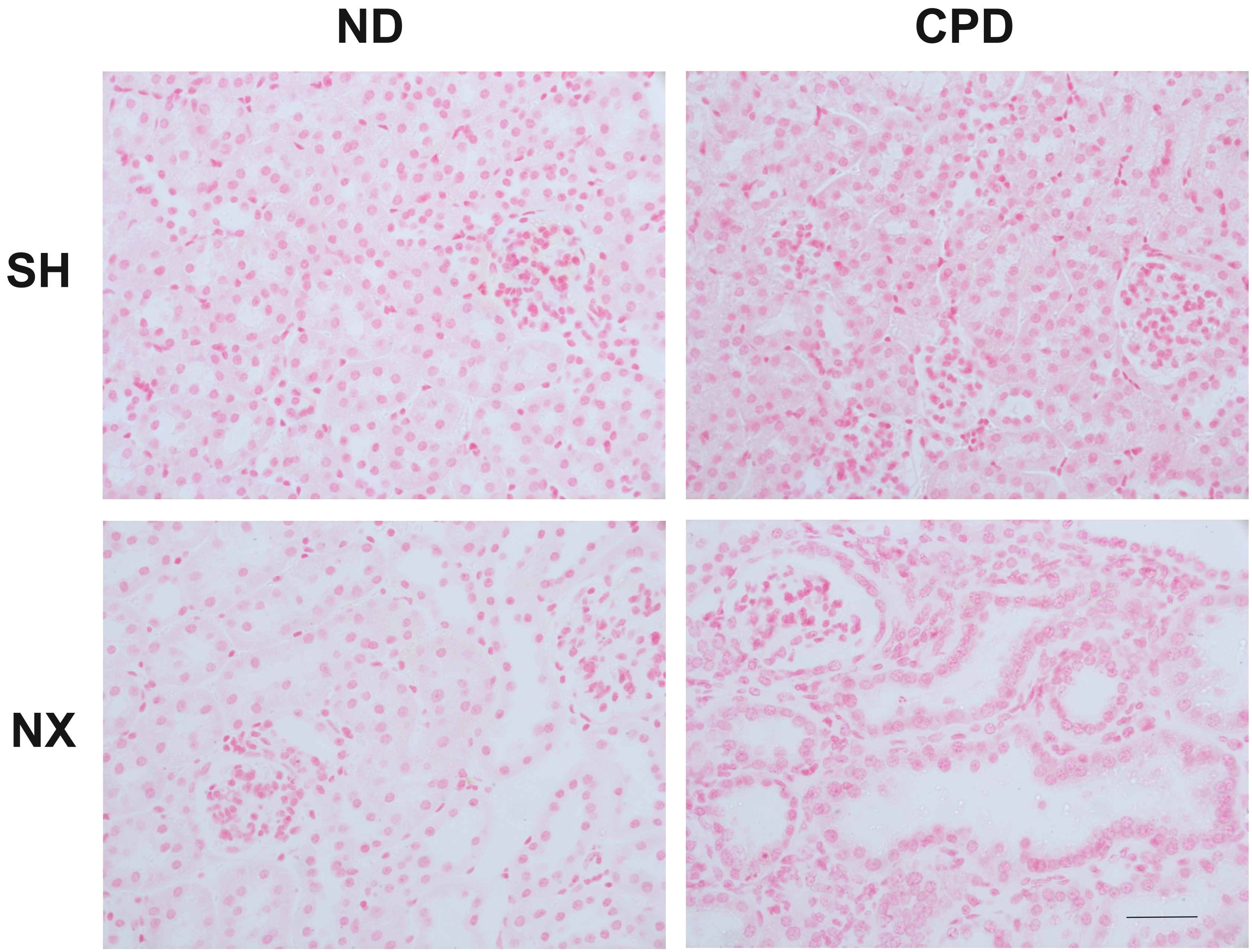

Supplementary Figure S1

Lack of calcium deposition in interstitial kidney tissue in 5/6-Nx mice.
Von Kossa staining revealed no calcium deposits in renal tissue of Sham-operated (SH) and 5/6-Nx (NX) C57BL/6 mice on ND or CPD, 12 weeks postsurgery. N= 6-12 mice per group.

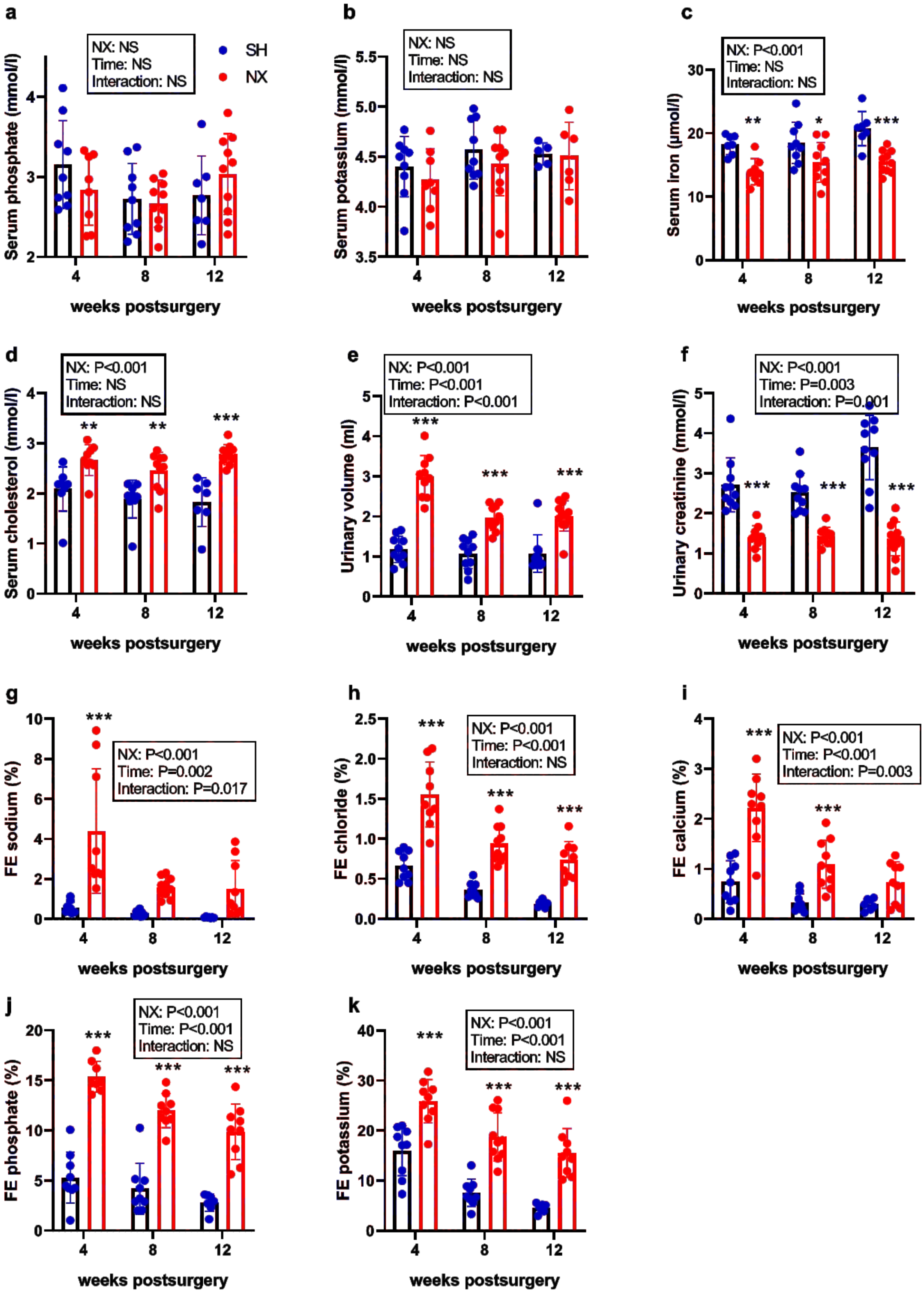

Supplementary Figure S2

Mineral metabolism in 5/6-Nx mice on CPD.
(a) Serum phosphate, (b) serum potassium, (c) serum iron, (d) serum cholesterol, (e) urinary volume, (f) urinary creatinine, and fractional urinary excretion (FE) of (g) sodium, (h) chloride, (i) calcium, (j) phosphate, and (k) potassium in Sham-operated (SH) and 5/6-Nx (NX) C57BL/6 mice on CPD, 4, 8 and 12 weeks postsurgery. N= 6-12 mice per group. Bars depict mean values ± SD. Data were analyzed using 2-way ANOVA, followed by Student's t-test. Insets show results of 2-way ANOVA. *, P<0.05; **, P<0.01; ***, P<0.001 vs. Sham at same time point by Student's t-test.

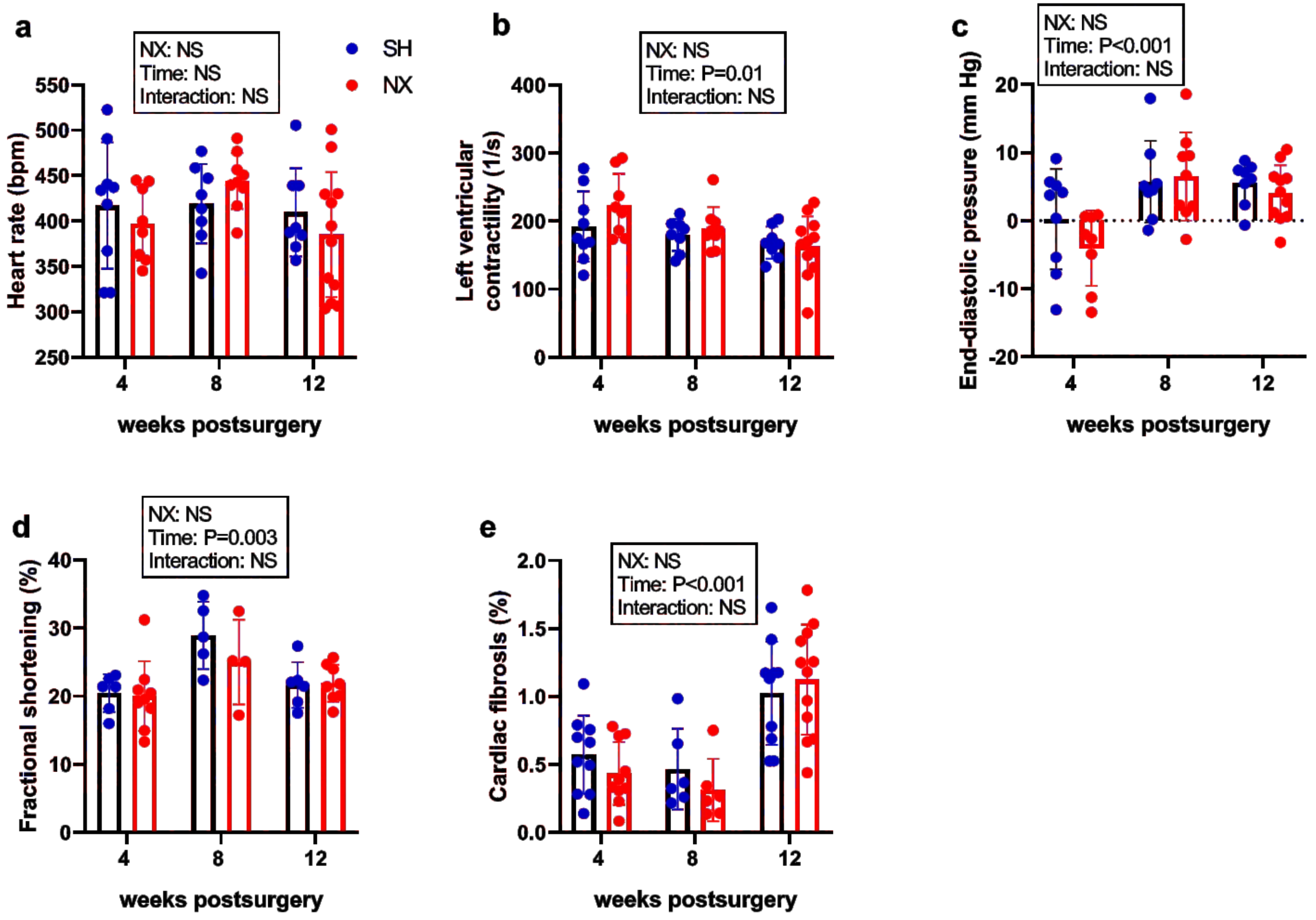

Supplementary Figure S3

Lack of left ventricular functional changes and fibrosis in 5/6-Nx mice on CPD.
(a) Heart rate, (b) left ventricular contractility, and (c) end-diastolic pressure measured by left ventricular catheterization, (d) fractional shortening measured by echocardiography, and (e) cardiac pircrosirius red-stained area in Sham-operated (SH) and 5/6-Nx (NX) C57BL/6 mice on CPD, 4, 8 and 12 weeks postsurgery. N= 6-12 mice per group. Bars depict mean values ± SD. Data were analyzed using 2-way ANOVA, followed by Student's t-test. Insets show results of 2-way ANOVA.

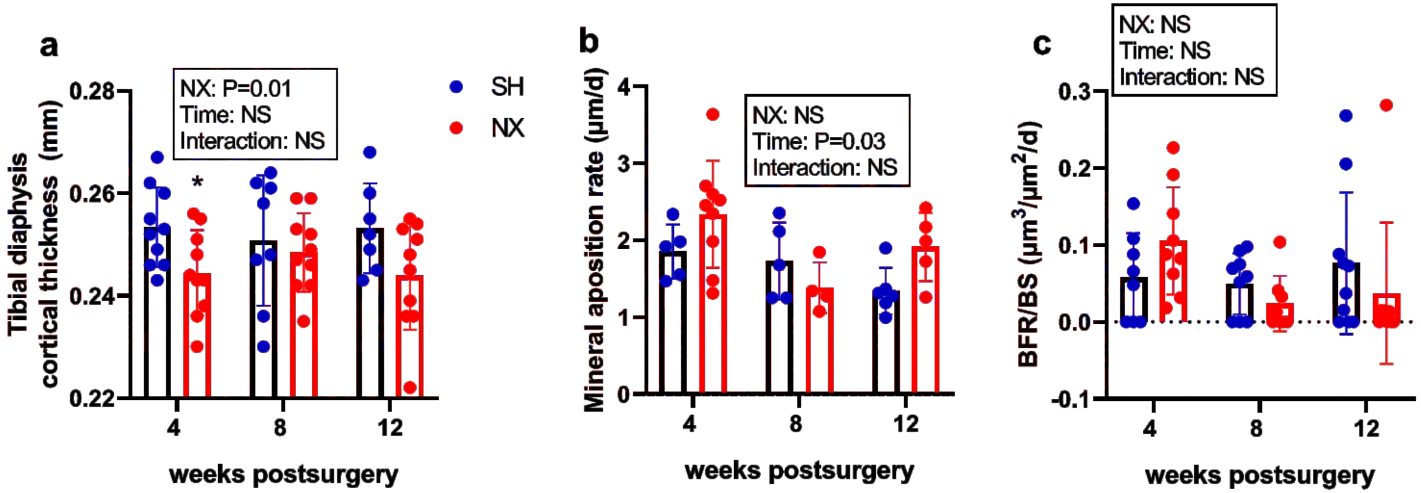

Supplementary Figure S4

Cortical thinning but unchanged bone formation rate in 5/6-Nx mice on CPD.
(a) Tibial diaphyseal cortical thickness measured by pQCT, as well as (b) mineral apposition rate (MAR) and (c) bone formation rate per bone surface (BFR/BS) measured in proximal tibial cancellous bone by histomorphometry in Sham-operated (SH) and 5/6-Nx (NX) C57BL/6 mice on CPD, 4, 8 and 12 weeks postsurgery. N= 4-10 mice per group. Bars depict mean values ± SD. Data were analyzed using 2-way ANOVA, followed by Student's t-test. Insets show results of 2-way ANOVA.
*, $P<0.05$ vs. Sham at same time point by Student's t-test.
